# The adenosine hypothesis of schizophrenia into its third decade: From neurochemical imbalance to early life etiological risks

**DOI:** 10.3389/fncel.2023.1120532

**Published:** 2023-03-14

**Authors:** Philipp Singer, Benjamin K. Yee

**Affiliations:** ^1^Roche Diagnostics International AG, Rotkreuz, Switzerland; ^2^Department of Rehabilitation Sciences, The Hong Kong Polytechnic University, Kowloon, Hong Kong SAR, China; ^3^Mental Health Research Centre, The Hong Kong Polytechnic University, Kowloon, Hong Kong SAR, China

**Keywords:** adenosine, DNA methylation, epigenetics, immune response, RNA editing, schizophrenia

## Abstract

The adenosine hypothesis of schizophrenia was conceptualized about two decades ago in an attempt to integrate two prominent theories of neurochemical imbalance that attribute the pathogenesis of schizophrenia to hyperfunction of the mesocorticolimbic dopamine neurotransmission and hypofunction of cortical glutamate neurotransmission. Given its unique position as an endogenous modulator of both dopamine and glutamate signaling in the brain, adenosine was postulated as a potential new drug target to achieve multiple antipsychotic actions. This new strategy may offer hope for improving treatment, especially in alleviating negative symptoms and cognitive deficits of schizophrenia that do not respond to current medications. To date, however, the adenosine hypothesis has yet led to any significant therapeutic breakthroughs. Here, we address two possible reasons for the impasse. First, neither the presence of adenosine functional deficiency in people with schizophrenia nor its causal relationship to symptom production has been satisfactorily examined. Second, the lack of novel adenosine-based drugs also impedes progress. This review updates the latest preclinical and clinical data pertinent to the construct validity of the adenosine hypothesis and explores novel molecular processes whereby dysregulation of adenosine signaling could be linked to the etiology of schizophrenia. It is intended to stimulate and revitalize research into the adenosine hypothesis towards the development of a new and improved generation of antipsychotic drugs that has eluded us for decades.

## Introduction

Adenosine is an endogenous purine nucleoside essential to the regulation of many biochemical and physiological processes including neurotransmission (Fredholm et al., 2005), bioenergetics (Fredholm et al., 2011), immune reaction (Haskó et al., 2005), and epigenetic processes (Williams-Karnesky et al., 2013). Disturbances to one or more of these processes have been linked to the etiology and/or symptom genesis of schizophrenia (Boison et al., 2012). In the mammalian brain, adenosine acts as a neural signaling molecule through the activation of four G-protein-coupled adenosine receptors: adenosine A1 receptor (ADORA1), adenosine A2A receptor (ADORA2A), adenosine A2B receptor (ADORA2B), and adenosine A3 receptor (ADORA3; Ribeiro et al., 2002; Fredholm et al., 2005; Chen J.-F. et al., 2014). Under physiological concentrations, adenosine stimulates primarily ADORA1 and ADORA2A that differ in function and expression patterns. In the mammalian brain, ADORA1 is expressed throughout the central neuronal system (CNS) with a high abundance in the neocortex, cerebellum, hippocampus, and the dorsal horn of the spinal cord. On the other hand, ADORA2A is enriched in striatal neurons with lower expression levels in the cerebral cortex, hippocampus, and thalamus (Ribeiro et al., 2002; Fredholm et al., 2005; Chen J.-F. et al., 2014). The general distribution of ADORA1 and ADORA2A is comparable between rodents and humans although the expression of ADORA2A in extra-striatal regions seems to be higher in humans than rodents (for a review see Fredholm et al., 2005).

**Figure 1:**
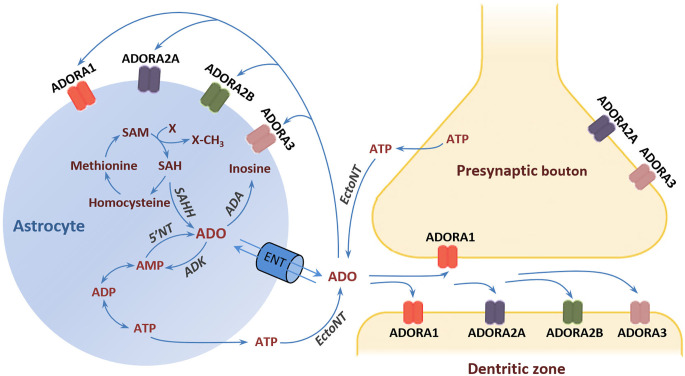
Adenosine receptors and metabolism. The major source of extracellular adenosine is the extracellular degradation of ATP and AMP by EctoNTs (Latini and Pedata, 2001; Pascual et al., 2005). Astrocytes can release ATP *via* vesicle exocytosis or hemichannels (Boison et al., 2010). In addition, adenosine can be directly released form astrocytes through ENTs following elevation of the intracellular adenosine concentration through hydrolysis of AMP and SAH by 5‘NT and SAH, respectively. The adenosine reuptake through ENTs is driven by the action of the intracellular enzyme ADK, which phosphorylates adenosine into AMP. ADK is considered the key regulator of extracellular adenosine under physiological conditions (Studer et al., 2006). An alternative metabolic route that comes into play under conditions of increased adenosine is the deamination of adenosine to inosine by the enzyme ADA (Lloyd and Fredholm, 1995). Adenosine exerts its effects through the activation of four types of G-protein-coupled receptors: ADORA1, ADORA2A, ADORA2B, and ADORA3 (Fredholm et al., 2005; Chen et al., 2014). *Abbreviations*: ADA, adenosine deaminase; ADK, adenosine kinase; ADORA1, adenosine A1 receptor; ADORA2A, adenosine A2A receptor; ADORA2B, adenosine A2B receptor; ADORA3, adenosine A3 receptor; AMP, adenosine monophosphate; ADP, adenosine diphosphate; ATP, adenosine triphosphate; 5’NT, 5’-nucleotidase; EctoNT, ectonucleotidase; ENT, equilibrative nucleoside transporter; SAH, S-adenosylhomocysteine; SAHH, S-adenosylhomocysteine hydrolase; SAM, S-adenosylmethionine.

Activation of ADORA1 inhibits the release of dopamine and glutamate, depresses neural excitability, and suppresses neuroplasticity (De Mendonça and Ribeiro, 1997, 2000; Dunwiddie and Masino, 2001; Ciruela et al., 2006). By contrast, ADORA2A activation facilitates the release of dopamine and glutamate in nerve terminals, and it is critically involved in the induction of long-term potentiation (LTP) in the hippocampus (Kessey and Mogul, 1997; Ciruela et al., 2006; Rebola et al., 2008). Moreover, ADORA1 and ADORA2A not only interact directly with each other but also with dopamine D_1_ and D_2_ receptors (D_1_Rs and D_2_Rs) and glutamate metabotropic receptors (mGluRs) by forming heteromeric receptor complexes (George et al., 2002; Ferré, 2008). For instance, the expression of ADORA1/D_1_R and ADORA2A/D_2_R heteromers expressed in the dendritic spines of striatal medium spiny neurons underlies the functional antagonism between adenosine and dopamine in the regulation of striatal output (Ferré, 2008). This antagonism at ADORA2A/D_2_R has led to the suggestion that stimulation of ADORA2A may confer antipsychotic potential through the suppression of D_2_R activity (Ferré et al., 1994). It is germane to the formulation of a recognizable adenosine hypothesis of schizophrenia in the beginning of the 2000’s (Lara et al., 2006) that went beyond the systemic purinergic model of schizophrenia (Lara and Souza, 2000). Specifically, the adenosine hypothesis postulates that brain adenosine deficiency can cause schizophrenia symptoms and the strengthening of adenosinergic signaling ought to be therapeutic. Such therapeutic approaches may also include intra- and extracellular enzymatic regulators of adenosine production and clearance in the CNS. One suggestion is to target the enzyme adenosine kinase (ADK) whose activity in astrocytes determines the extracellular adenosine concentration in the adult brain (Studer et al., 2006; Etherington et al., 2009). The conversion of adenosine into adenosine monophosphate (AMP) by ADK drives the influx of adenosine from the extracellular space *via* bidirectional equilibrative nucleoside transporters (ENTs). Similarly, the production and clearance of adenosine may also be manipulated by interference with ectonucleotidases (EctoNTs) that break down adenosine triphosphate (ATP) to adenosine, and deaminase (ADA) that degrades adenosine to inosine (Bontemps et al., 1983; Etherington et al., 2009; Figure 1).

Preclinical animal models of schizophrenia have been instrumental in the specification of relevant cellular and molecular mechanisms by which adenosine dysregulation can give rise to schizophrenia-like behavioral and cognitive abnormalities (for reviews see Boison et al., 2012; Rial et al., 2014). The translation of these proof-of-concept studies to the bedside, however, has been slow. One roadblock is a lack of consensus for adenosine deficiency in schizophrenia, and it is timely for an update of the latest clinical evidence. Secondly, there is a lack of novel adenosine modulating drugs in the pipeline available for evaluation. Innovative drug mechanisms are necessary in the search of truly revolutionary treatment of schizophrenia. In this article, we aim to review evidence in support of the adenosine hypofunction hypothesis emerged from the latest clinical studies. Finally, we explore adenosine-dependent immune and epigenetic processes from the etiological perspective of schizophrenia in an attempt to seek novel insights to guide future antipsychotic development.

## Adenosine hypofunction in schizophrenia

### Adenosine kinase expression levels are unaltered in schizophrenia

Central to the adenosine hypothesis is that a reduction in central adenosine levels is the root cause of the specific hyper- and hypo-function of the dopamine and glutamate neurotransmission, respectively, which underlies symptoms production in schizophrenia (Boison et al., 2012; Rial et al., 2014). In keeping with this hypothesis, reduced extracellular adenosine in the brain of transgenic mice due to neuronal over-expression of ADK yielded concomitant alterations in dopaminergic and glutamatergic functions (Yee et al., 2007). The motor stimulant response to NMDA receptor blockade was enhanced, the mutant mice exhibited severe learning deficits, and adenosine augmentation was able to normalize behavior in these mutants (Shen et al., 2012). However, a recent post-mortem study failed to detect any significant change in the expression of ADK mRNA and protein levels in the dorsolateral prefrontal cortex (DLPFC) or anterior cingulate cortex of schizophrenia subjects compared with age- and sex-matched healthy controls (Moody et al., 2020). Cell-type specific measurement of ADK gene expression in enriched populations of astrocytes and pyramidal neurons in DLPFC also did not reveal any differences between patients and controls (O’Donovan et al., 2018). Likewise, ADK mRNA expression in peripheral blood showed no alteration in patients with schizophrenia (Kimura et al., 2018). Finally, *in silico* analysis of post-mortem expression of ADK using microarray and RNAseq databases, did not identify any abnormalities of ADK expression in frontal cortices in people with schizophrenia (Moody et al., 2020). These consistent null findings of ADK expression in the schizophrenic brain suggest that an elevation of ADK protein expression is not a likely cause of the proposed adenosine hypofunction in schizophrenia. If anything, a rare case with a copy number variant of the *ADK* gene has been discovered in a cohort of 1,699 schizophrenia patients, who had extremely low ADK levels in the blood (Kimura et al., 2018). Hence, we may not rule out completely that ADK expression is altered in a small subset of patients. But the reduction in ADK expression in this case would be expected to elevate ambient adenosine levels, which would contradict with the expectations of the adenosine hypofunction hypothesis.

One limitation of existing clinical studies is the lack of complementary protein assays measuring the enzymatic activity of ADK since the protein expression level alone does not necessarily predict overall enzyme activity. Other factors may influence ADK activity in schizophrenia patients such as the local availability of its co-factor Mg^2+^ (Spychala et al., 1996; McNally et al., 1997), the relative expression of the two ADK isoforms (McNally et al., 1997), or the occurrence of any mutations altering the active site, the substrate- or co-factor binding site, or the overall folding of the enzyme.

### Elevated adenosine deaminase activity in schizophrenia

While ADK expression appears largely unaffected in schizophrenia, there is evidence that other metabolic regulators of adenosine might be affected with consequential reduction in ambient adenosine levels in the brain. A potential candidate is ADA, an enzyme that metabolizes adenosine to inosine (Figure 1). Increased ADA gene expression levels have been detected in pyramidal neurons of the DLPFC of schizophrenia patients compared with controls (O’Donovan et al., 2018). The inability of chronic haloperidol to modulate the expression of ADA in pyramidal neurons in rats (O’Donovan et al., 2018) suggests that the efficacy of typical antipsychotic drugs to suppress psychotic symptoms may not be readily attributed to elevated ADA protein levels in the brain.

A rare polymorphism of ADA with lower enzymatic activity was found to be less frequent in a Brazilian cohort of schizophrenia patients (Dutra et al., 2010). The degradation of adenosine by ADA may be more efficient in these patients, which is expected to lower adenosine availability. However, this specific polymorphism may at best be linked to 5% of patients (Dutra et al., 2010). Evidence of increased ADA activity in schizophrenia patients treated with antipsychotic drugs has been obtained by measuring the enzymatic activity of serum ADA (Brunstein et al., 2007). A subsequent study also reported increased serum protein levels of ADA in drug-naïve first-episode patients in comparison with healthy volunteers (Sasidharan et al., 2017). These authors went on to suggest using elevated serum ADA levels as a biomarker of schizophrenia. However, the elevation of this system marker may still be linked to the type of antipsychotic medication. Patients treated with clozapine showed higher serum ADA activity than patients treated with haloperidol (Ghaleiha et al., 2011). Treatment responsiveness to clozapine was also found to correlate positively with serum ADA levels. This has been attributed to a compensatory response to the cellular production of adenosine stimulated by clozapine (Lara et al., 2001; Ghaleiha et al., 2011). Hence, there are some grounds to suspect an association of elevated ADA activity with schizophrenia in the direction as predicted by the adenosine hypofunction hypothesis.

Regarding the psychotropic effects of ADA inhibition, an antidepressant-like effect has been reported following administration of erythro-9-(2-hydroxy-3nonyl) adenine (EHNA) in a mouse model of behavioral despair (Kaster et al., 2013). This finding reinforces earlier reports by the same research group showing that administration of exogenous adenosine yielded a similar antidepressant effect (Kaster et al., 2004, 2005), and hence the suggestion that negative symptoms of schizophrenia, such as avolition and anhedonia, may be ameliorated by increasing adenosine levels. However, this hypothesis is contradicted by prior studies showing the exact opposite results: namely, exogenous adenosine could exacerbate depression-like behavior (Porsolt et al., 1977; Kulkarni and Mehta, 1985). A more complex picture emerges when the reported effects of ADORA1 and ADORA2A agonists and antagonists in preclinical models of depressive behavior are taken into consideration (see Table 1). Amidst the mixed outcomes, the behavioral pharmacology data tend to align with the interpretation that inhibition, rather than stimulation, of adenosine receptors, may be more likely to yield an antidepressant profile.

**Table 1:** Effects of adenosinergic drugs in animal models relevant to the negative symptoms of schizophrenia.

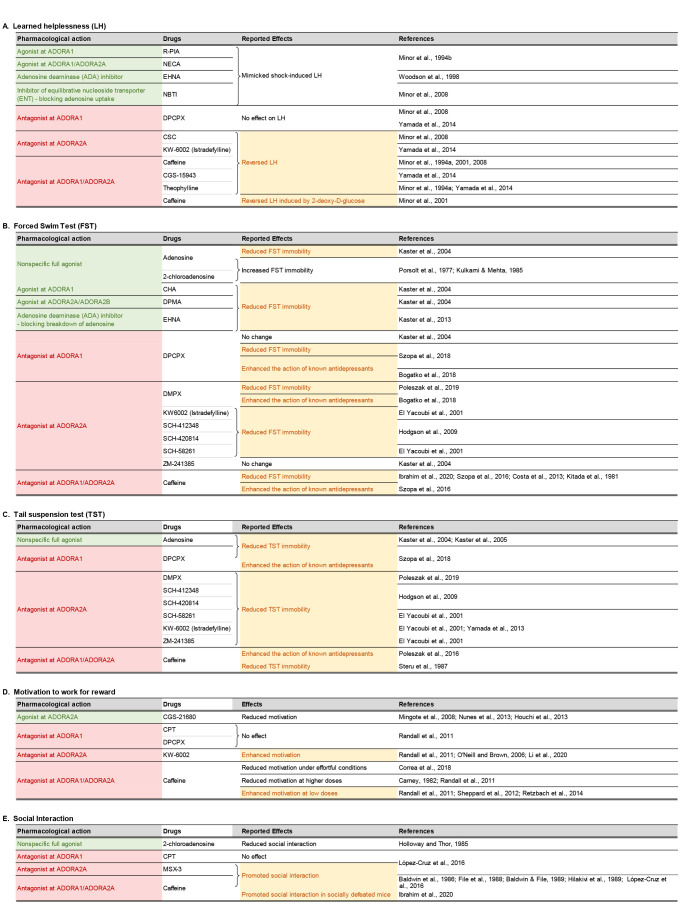

Blockade rather than stimulation of ADORA2A and ADORA1 is associated with antidepressant properties in animal models of behavioral despair and avolition: (**A**) learned helplessness, (**B**) forced swim test, and (**C**) tail suspension test (Duman, 2010; Yee and Singer, 2013). (**D**) Another class of animal models focuses on motivation in which animals are required to work to obtain a positive reinforcement (e.g., a food reward). The motivation to work for food reward is reduced by the ADORA2A agonist, CGS-21680, but the effect was only apparent under high but not low-effort requirements (Mingote et al., 2008). This effect has been attributable to the stimulation ADORA2As in the nucleus accumbens, which is expected to inhibit the activation of accumbal D_2_Rs (Mingote et al., 2008; Nunes et al., 2013). In agreement, selective blockade of ADORA2A enhances motivation and performance under high-effort requirements whereas blockade of ADORA1 has no notable effect on the motivation to work for rewards (Randall et al., 2011). (**E**) Another notable negative symptom is the withdrawal from social engagement and interaction. The limited data show that blockade of ADORA2A but not ADORA1 enhances social interaction while caffeine tends to reduce social interaction. Overall, available preclinical data suggest that blockade of ADORA2A may improve negative symptoms in schizophrenia including psychomotor slowing, apathy, anergia, avolition, and social withdrawal. These positive findings are highlighted by a yellow background in the column of “Reported Effects”. *Keys*: Pharmacological actions expected to up- and down-regulate adenosinergic signals are in green and red, respectively. *Abbreviations*: ADORA1, adenosine A1 receptor; ADORA2A, adenosine A2A receptor; ADORA2B, adenosine A2B receptor; CHA, N^6^-cyclohexyl-adenosine; CPT, 8-Cyclopentyltheophylline; CSC, 8-(3-Chlorostyryl)caffeine; DPCPX, Dipropylcyclopentylxanthine (8-Cyclopentyl-1,3-dipropylxanthine); DMPX, 3,7-dimethyl-1-propargylxanthine; DPMA, N(6)-[2-(3,5-dimethoxyphenyl)-2-(2-methylphenyl)-ethyl]adenosine; EHNA, Erythro-9-(2-hydroxy-3-nonyl)adenine; NBTI, Nitrobenzylthioinosine; NECA, 5’-N-Ethylcarboxamidoadenosine; R-PIA, (-)-R-(N6-phenylisopropyl)adenosine. IUPAC Names of coded compounds are: **CGS-15943**: 9-chloro-2-(furan-2-yl)-[1,2,4]triazolo[1,5-c]quinazolin-5-amine; **CGS-21680**: 3-4-[2-(6-Amino-9-[(2R,3R,4S,5S)-5-(ethylcarbamoyl)-3,4-dihydroxyoxolan-2-yl]-9H-purin-2-ylamino)ethyl]phenylpropanoic acid; **KW-6002** (a.k.a. Istradefylline): 8-[(E)-2-(3,4-dimethoxyphenyl)ethenyl]-1, 3-diethyl-7-methylpurine-2,6-dione; **MSX-3**: 3,7-Dihydro-8-[(1E)-2-(3-methoxyphenyl)ethenyl]-7-methyl-3-[3-(phosphonooxy)propyl]-1-(2-propynyl)-1H-purine-2,6-dione Disodium Salt; **SCH-412348**: 10-[2-[4-(2,4-difluorophenyl)piperazin-1-yl]ethyl]-4-(furan-2-yl)-3,5,6,8,10, 11-hexazatricyclo[7.3.0.02,6]dodeca-1(9),2,4,7,11-pentaen-7-amine; **SCH-420814**: 4-(furan-2-yl)-10-[2-[4-[4-(2-methoxyethoxy)phenyl]piperazin-1-yl]ethyl]-3,5,6,8,10,11-hexazatricyclo[7.3.0.02,6]dodeca-1(9),2,4,7,11-pentaen-7-amine; **SCH-58261**: 4-(furan-2-yl)-10-(2-phenylethyl)-3,5,6,8,10,11-hexazatricyclo[7.3.0.02,6]dodeca-1(9),2,4,7,11-pentaen-7-amine; **ZM-241385**: 4-(2-[7-Amino-2-(furan-2-yl)[1,2,4]triazolo[1,5-a][1,3,5]triazin-5-yl]aminoethyl)phenol.

As pre- and postsynaptic ADORA1s and ADORA2As play different roles in the regulation of neural signaling (Fredholm et al., 2005; Chen J.-F. et al., 2014; Ferré et al., 2023), the contradictory observations could be related to differences in the intrinsic preference for pre- or postsynaptic receptor populations among the tested compounds summarized in Table 1. It has been shown that ADORA2A antagonists are distinguishable according to their preference for pre- vs. postsynaptic ADORA2As in the striatum (Orru et al., 2011). Hence, it may be conceivable that competing pre- and postsynaptic actions of pharmacological interventions targeting ADORA1 and ADORA2A may underlie the mixed results observed in animal models of negative symptoms although the precise mechanisms remain elusive.

One possibility that the potential antidepressant effect of exogenous adenosine may not be faithfully mimicked by adenosine receptor agonists is that exogenous adenosine also increases the production of inosine (Figure 1), which also exhibited an anti-depressant profile by itself in preclinical evaluation, including the forced swim test and the tail suspension test (Kaster et al., 2013; Muto et al., 2014; Cunha et al., 2015; Gonçalves et al., 2017; Yuan et al., 2018). The precise mechanism underlying the antidepressant effects of inosine remains unclear, but it may not be entirely independent of adenosine receptor activity because ADORA1 and ADORA2A antagonists could reportedly nullify the antidepressant-like effect of inosine (Kaster et al., 2013).

The behavioral significance of the ambient levels of adenosine and inosine may best be explored further by correlative analysis using *in vivo* measures. Enzyme-linked microelectrode arrays allow the correlation of real-time local extracellular adenosine concentration with on-going behavior (Hinzman et al., 2015). Similar correlative analysis could well be extended to clinical studies, as various methods for *in vivo* measurement of the adenosine concentration in human blood or interstitial tissue are now available (Ramakers et al., 2008).

### Reduced EctoNT activity and expression in schizophrenia is linked to adenosine deficiency

EctoNTs refer to a family of enzymes responsible for the sequential hydrolysis of ATP to adenosine: ATP → ADP → AMP → adenosine (Yegutkin, 2008; Yegutkin and Boison, 2022). Two post-mortem studies in schizophrenia patients have reported a reduction of EctoNT activity in the striatum (Aliagas et al., 2013) and a downregulation of EctoNT mRNA expression in astrocytes in the DLPFC (O’Donovan et al., 2018). Such changes are expected to reduce ambient adenosine levels in keeping with the adenosine hypofunction hypothesis of schizophrenia. However, preclinical models do not lend support for a causal link of reduced EctoNT expression to symptom production. Instead, there is evidence suggesting otherwise.

One EctoNT that has attracted more attention is CD73. It is the rate-limiting enzyme regulating adenosine formation derived from the catalysis of ATP (Zimmermann et al., 2012). Genetic deletion of CD73 in mice blunted the development of amphetamine sensitization and improved working memory performance (Augusto et al., 2013; Zlomuzica et al., 2013). While the former phenotype is indicative of a resilience against mesolimbic hyperdopaminergic signaling linked to the production of positive symptoms in schizophrenia (Laruelle, 2000), the later pro-cognitive phenotype is opposite to the memory impairment in schizophrenia attributed to cortical dopamine deficiency (Luvsannyam et al., 2022). One explanation of the anti-hyperdopaminergia phenotype is in terms of the colocalization of ADORA2A and CD73 in striatal neurons, whereby CD73 is the main source of adenosine that stimulates ADORA2A (Augusto et al., 2013). It provides a mechanism for striatal ADORA2A auto-stimulation. Although its normal function remains elusive, ADORA2A auto-stimulation would be severely undermined by the attenuation of CD73 expression in these neurons. ADORA1-mediated signaling, on the other hand, appears unaffected by the deletion of CD73 (Zhang D. et al., 2012), suggesting that the constitutive loss of CD73 function is not capable of causing a brain-wide reduction of adenosine. It follows that pharmacological inhibition of CD73 could be a viable alternative to downregulate striatal ADORA2A activity that contributes to the development of sensitization of mesolimbic dopamine signaling. In line with this suggestion, striatum-specific deletion of ADORA2A can similarly attenuate sensitization to amphetamine and enhance working memory—phenotypes that are also seen after global or forebrain ADORA2A deletion (Chen et al., 2003; Bastia et al., 2005; Zhou et al., 2009; Wei et al., 2011). At least one ADORA2A antagonist, KW6002, has been shown to possess a pro-cognitive effect in spatial working memory in both rodents and non-human primates (Li et al., 2018).

Resilience against drug-induced mesolimbic hyperdopaminergia and enhanced working memory performance consistently observed in the animal models above suggest that downregulating the expression of EctoNTs may ameliorate rather than exacerbate the positive symptoms (attributable to hyperdopaminergia) and working memory impairment commonly experienced in schizophrenia patients. It directly contradicts the suggestions that reduced expression of EctoNTs in schizophrenia patients contributes to the production of schizophrenia symptoms. One alternative interpretation is to attribute the reduction of EctoNT to antipsychotic medication. Yet, it is known that the typical antipsychotic drug, haloperidol, could not induce such compensatory changes in normal rats (O’Donovan et al., 2018). Whether atypical antipsychotics, such as clozapine, have the ability to suppress EctoNT expression remains to be elucidated.

The apparent discrepancy between the post-mortem findings and preclinical models poses some difficulty with respect to any attempt to associate a brain-wide adenosine deficiency with the pathogenesis of schizophrenia. It suggests that adenosine deficiency in distinct brain regions must be taken into consideration, and the challenge is to specify the regions and/or circuits whereby adenosine deficiency could induce schizophrenia-like aberrations in behavior, moods, and thinking. At the same time, we may need to be open for the possibility that upregulation of adenosinergic activity in some regions are linked to symptom production whereas downregulation of adenosine activity in other brain regions produces antipsychotic effects.

### Nucleoside transporters expression is reduced in schizophrenia

Transmembrane trafficking of adenosine is regulated by ENTs (see Figure 1). Hence, the functional significance of ENT blockade on the balance of intra- and extracellular adenosine would be strongly dependent on the local adenosinergic environment (Dunwiddie and Masino, 2001; Latini and Pedata, 2001). A reduction of ENT1 expression in the superior temporal gyrus has been reported in schizophrenia patients (Shan et al., 2012). A similar reduction, specific to pyramidal neurons in the DLPFC, was also found (O’Donovan et al., 2018). O’Donovan et al. (2018) interpreted the reduction in neuronal ENT1 expression as a compensatory response to lower extracellular adenosine release (*via* ENT1s) by active spiking neurons. The release of adenosine by active spiking neurons is expected to suppress neural excitation *via* stimulation of ADORA1, which constitutes a part of the negative feedback loop to prevent over-excitation (Lovatt et al., 2012). The delicate balance of this feedback may be disturbed by the downregulation of ENT1 expression reported in the schizophrenic brain. Yet, one must be cautious before concluding if impaired adenosine trafficking *via* ENTs is intrinsic to the disease. Clozapine may also indirectly lead to a compensatory downregulation of ENT because of its ability to boost adenosine production, although conventional antipsychotic drugs, like haloperidol, had no such ability as demonstrated in healthy rats (O’Donovan et al., 2018).

### Summary of enzymatic regulation of adenosine in schizophrenia

With the exception of ADK, there is no shortage of evidence for an elevation of ADA activity, and a downregulation of EctoNTs as well as ENTs, which may all contribute to an extracellular environment deficient of adenosine in the schizophrenic brain. Any attempt to answer how such a neurochemical environment could generate multiple schizophrenia symptoms, however, is necessarily speculative and far from simple. The extent to which normalization of such adenosine deficiency may be therapeutic must be tested empirically in preclinical models, and clinical trials that will include serum measures of relevant enzymatic regulators to enable a correlation with severity and types of symptoms. In addition, a systematic evaluation of the effects of antipsychotic drugs on the expression of ADA, EctoNT, and ENT may help separate possible adenosinergic disturbances intrinsic to schizophrenia from those that may be caused by antipsychotic mediation.

### The expression of adenosine receptors in the schizophrenia brain

Reports of elevated ADORA2A expression in the hippocampus (Kurumaji and Toru, 1998) and striatum (Hwang et al., 2013) of patients with schizophrenia have been interpreted as a compensatory upregulation in response to reduced adenosine availability (Boison et al., 2012; Rial et al., 2014). However, there is increasing evidence that the compensatory changes were attributable to antipsychotic medications. A positive correlation between antipsychotic drug dosage and striatal ADORA2A density was identified in post-mortem materials (Kurumaji and Toru, 1998; Deckert et al., 2003). The increase of ADORA2A gene expression in platelets only emerged after 6 weeks of antipsychotic treatment but could not be replicated in drug-free patients (Zhang J. et al., 2012). Furthermore, post-mortem analysis of prefrontal cortex samples revealed no differences in the expression levels of ADORA2A gene or protein between schizophrenia patients and healthy controls, nor between drug-free and antipsychotic-treated patients (Urigüen et al., 2009). Likewise, the only positron emission tomography (PET) imaging study to date has failed to identify an increase in ADORA2A levels in medicated patients compared with healthy controls (Marques et al., 2022). Although, this study may be limited by a relatively small sample size (12 patients vs. 13 controls) and the restriction to male subjects. A larger cohort of samples, inclusive of both genders, would be warranted in any future PET studies.

Using an AlphaLISA-based immunoassay, Valle-León et al. (2021) reported a pronounced reduction in the formation of ADORA2A-D_2_R heterodimers against a background of an elevated expression of ADORA2A and D_2_R protein in the caudate nucleus of schizophrenic subjects compared with post-mortem tissues obtained in healthy controls. The authors went on to show in a parallel murine experiment that the psychomimetic drug, phencyclidine (an NMDA receptor antagonist) could induce a similar reduction in ADORA2A-D_2_R heterodimerization in the striatum, and antipsychotic drugs (haloperidol or clozapine) could attenuate the hypo-heterodimerization. This led Valle-León et al. (2021) to speculate that a drop in striatal ADORA2A-D_2_R heterodimerization may be a mechanistically relevant biomarker of schizophrenia and drugs that can facilitate the formation of ADORA2A-D_2_R heterodimers may possess therapeutic potential.

Regarding the expression of ADORA1 in schizophrenia, one study reported comparable expression in the striatum in patients and controls (Villar-Menéndez et al., 2014). Another post-mortem study detected a reduction in ADORA1 expression in the prefrontal cortex, but it was attributed to antipsychotic medication (O’Donovan et al., 2018).

We should not completely exclude the post-mortem study reporting a reduction of striatal ADORA2A expression in 50% of male patients with schizophrenia (Villar-Menéndez et al., 2014). The reduction was apparently not statistically linked to antipsychotic use. The levels of ADORA2A downregulation further correlated with the severity of motor disturbances but not with respect to positive, negative, or general subscores in the Positive and Negative Syndrome Scale (PANSS). This lack of association with positive and cognitive symptoms does not correspond well to the genetic knockout mouse models summarized above. Whether these patients’ prominent motor disturbance might benefit from adjunctive ADORA2A agonist treatment remains to be tested. If so, there may exist specific symptoms in schizophrenia patients that PET imaging could help identify for personalized symptom control.

In short, it remains doubtful if schizophrenia is associated with an upregulation of brain ADORA2A or ADORA1 expression that one may infer as a functional compensation to an underlying adenosine deficiency intrinsic to the disease without fear from confounding influences due to antipsychotic medication. This may be taken as a crucial lack of support for the adenosine hypofunction hypothesis. Here, we may turn to the advent of PET imaging tracers specific to ADORA2A or ADORA1 (e.g., Khanapur et al., 2014; Mishina and Ishiwata, 2014; Barret et al., 2015; Guo et al., 2018; Li et al., 2019; Joya et al., 2021; Lai et al., 2021). The first PET study in schizophrenia was only performed recently with the ADORA2A radioligand [^11^C]SCH442416, which failed to differentiate medicated chronic schizophrenia patients from healthy controls (Marques et al., 2022). The extension of similar PET studies to unmedicated and acute patients with a longitudinal design will provide decisive data. The use of radiolabeled antibodies against enzymes such as ADA, ADK, or EctoNTs in PET imaging can shed more light on the adenosine hypothesis. A radiolabeled human antibody for the EctoNT, CD73, has been successfully tested as an imaging probe in mice with clear potential for translation to humans (Sudo et al., 2020). Neuroimaging in patients can also examine if alterations in the expression of specific adenosine receptors and regulatory proteins may be segregated into symptom clusters. Such clinically relevant data will clarify if the therapeutic potential of drugs targeting ADORA2A or ADORA1 (as discussed above and reviewed next) should be viewed as corrective in nature or primarily in terms of symptom suppression.

## Current adenosine-based treatment approaches

### Existing adenosine augmentation therapy offers limited therapeutic efficacy

The adenosine hypofunction hypothesis predicts that drugs that increase extracellular adenosine could be effective antipsychotics. To date, three compounds have been evaluated. They are mechanistically distinct from each other:

• **Allopurinol** is a xanthine oxidase inhibitor that slows down the degradation of purines and thereby elevates extracellular purines including adenosine (Hirota and Kishi, 2013; Rogosnitzky et al., 2016). Three small-scale single-site randomized controlled trials have reported improved clinical outcomes when allopurinol was administered as add-on to conventional antipsychotics, with more improvement in positive and total PANSS scores than placebo add-on (Akhondzadeh et al., 2005; Brunstein et al., 2005; Dickerson et al., 2009). However, a subsequent larger multi-center trial failed to substantiate these claims (Weiser et al., 2012). Another recent case report had hinted at the potential of adjuvant allopurinol to achieve long-term reduction in aggressive behavior in a female patient with a persecutory delusion (Miyauchi, 2021), but the generalization of this singular observation must be evaluated in clinical trials.
• **Dipyridamole** increases extracellular adenosine by blocking adenosine reuptake (Hirota and Kishi, 2013; Rogosnitzky et al., 2016). When added to haloperidol, dipyridamole led to greater reduction in the total, positive, and general PANSS scores compared with placebo add-on, but dipyridamole was ineffective against negative symptoms (Akhondzadeh et al., 2000). In one small-scale monotherapy study, dipyridamole did not yield any notable therapeutic efficacy (Wonodi et al., 2011).
• **Propentofylline** is a xanthine derivative that increases the extracellular level of adenosine by blocking its reuptake and inhibiting cAMP degradation (Salimi et al., 2008). One placebo-controlled study evaluated propentofylline added to risperidone in chronic patients and reported improvement in both positive symptoms and general psychopathology (Salimi et al., 2008).

A consensus certainly cannot be derived from this handful of studies, especially when most of them lacked sufficient statistical power. Hence, they must be interpreted with caution. When the data across the three drugs were pooled for a meta-analysis, the overall effect size was small and the therapeutic efficacy of the three drugs as adjuvants to conventional antipsychotics was limited largely to positive symptoms (Hirota and Kishi, 2013). No benefits in negative or cognitive symptoms were evident where the medical need is most acute. A more definitive evaluation of the potential of adenosine augmentation therapy, however, would require the development of more selective compounds, since allopurinol, dipyridamole, and propentofylline are all associated with effects beyond the biosynthesis and reuptake of adenosine.

A recent Finnish longitudinal study showed that allopurinol and dipyridamole add-on treatment reduced the re-hospitalization risk in a large sample (*N* = 61,889) of patients with schizophrenia over a follow-up period between 1996 and 2017 (Lintunen et al., 2021). The benefits were more pronounced in younger patients suggesting that early treatment could help improve long-term functioning.

Thus, existing evidence for therapeutic efficacy of adjunctive adenosine augmentation therapy in schizophrenia is largely limited to positive symptoms. Its clinical value remains questionable since it may neither replace existing antipsychotic drugs nor address cognitive and negative symptoms. More clinical trials would be necessary for a more critical analysis, including the long-term rehabilitation and quality-of-life outcomes. Furthermore, larger monotherapy trials would be needed to identify potential antagonistic interactions between adenosinergic drugs and current antipsychotics that may limit the potential therapeutic efficacy of adenosine augmentation therapy. Another important consideration is the possibility of a ceiling effect when used in combination with clozapine given the latter’s ability to promote adenosine production. To be a viable alternative monotherapy, comparison against standard antipsychotic mediation must at least show comparable, if not superior, clinical efficacy and reduced side effects or higher tolerability.

### May caffeine benefit schizophrenia patients through arousal?

Excessive intake of caffeine, a mixed ADORA1/ADORA2A antagonist, can induce psychosis in healthy individuals as well as exacerbate positive symptoms in schizophrenia patients (Lucas et al., 1990; Nehlig et al., 1992; Hughes et al., 1998). This is consistent with the adenosine hypofunction hypothesis. The most plausible mechanism for caffeine-induced psychosis is the potentiation of the mesolimbic dopaminergic neurotransmission. Reports of higher coffee consumption in people with schizophrenia above the general population are consistent with their higher consumption of stimulant drugs (Hughes et al., 1998; Strassnig et al., 2006). If such behavior constitutes a form of self-medication, then dopamine is the most likely common pathway. Claims that acute caffeine could improve mood in the small double-blind placebo-controlled study (*N* = 13) by Lucas et al. (1990) is likely an exaggeration.

The common belief that caffeine can improve cognitive performance has received some support. Acute or chronic caffeine consumption in healthy people has been reported to improve semantic verbal fluency (Vercambre et al., 2013), visual memory (Borota et al., 2014), processing speed (Mackay et al., 2002), working memory (Smith, 2002, 2013; Smillie and Gökçen, 2010), and memory consolidation (Borota et al., 2014)—all of which are deficient in varying degrees in schizophrenia. Interestingly, a report of caffeine-induced improvement in the detection of subtle differences between memorized items (Borota et al., 2014) has been attributed to enhanced pattern-separation between stored representations in distributed memory networks—the deficiency of which has been linked to delusional thoughts and other psychotic symptoms in schizophrenia (Tamminga et al., 2010).

The possibility that patients with schizophrenia consume caffeine as a means to improve their cognitive function has been explored by Núñez et al. (2015). They reported that higher daily caffeine intake predicted better performance in semantic fluency, processing speed, working memory, and visual memory, but such effects were only observed in male patients. Neither the potential benefit to schizophrenia cognitive symptoms nor the gender difference reported is anticipated by the adenosine hypothesis. However, the observations are consistent with animal studies (see previous sections) showing that blockade of ADORA2A (rather than ADORA1) is associated with pro-cognitive efficacy including enhanced working memory performance.

However, the possibility that caffeine increases arousal rather than enhances the cellular processes underlying learning and memory has been raised (Nehlig et al., 1992; Nehlig, 2010). If so, its therapeutic window may be limited and vary substantially from person to person and personalized dosage may be necessary. It would also be hard to discriminate relief of symptoms from the promotion of wakefulness by caffeine (Landolt et al., 1995). Therefore, the use of caffeine runs the risk of exacerbating sleep disturbances, including insomnia, in schizophrenia (Monti et al., 2013).

## Etiological perspectives and future treatment strategies

We have summarized the critical evidence that supports as well as poses difficulties to the adenosine hypofunction hypothesis, including the clinical status of adenosine-based treatment approaches. The emphasis has been primarily on the possible mechanism of symptom production (i.e., face and construct validity) and symptom suppression (predictive validity). Next, we consider adenosine as a potential modulator from the neurodevelopmental perspective on the etiology of schizophrenia.

### Adenosine plays a role in the mediation of environmental risks factors for schizophrenia

Schizophrenia has a multifactorial etiology. A myriad of genetic and environmental predisposing risk factors jointly derails brain development from its normal path resulting in multiple brain functional disturbances responsible for the constellation of symptoms and disease progression that is recognizable and diagnosed as schizophrenia (Landek-Salgado et al., 2016; Millan et al., 2016; Owen et al., 2016; Prata et al., 2019; Richetto and Meyer, 2021). While genetic anomalies underlie the heritable component of schizophrenia, environmental factors may contribute *via* epigenetic processes (Tsankova et al., 2007; Peedicayil and Grayson, 2018). In fact, the majority of epigenetic alterations in schizophrenia are believed to originate from exposure to adverse environmental events during vulnerable periods in prenatal or perinatal life (Rutten and Mill, 2009; Grayson and Guidotti, 2013; Morishita et al., 2015; Nestler et al., 2016; Richetto and Meyer, 2021).

### Adenosine protects from inflammatory damage during an infection

**Figure 2:**
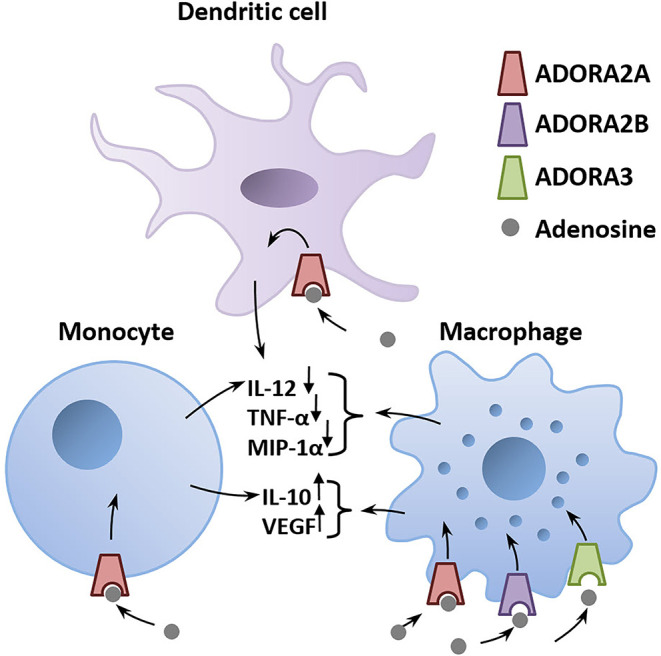
Adenosine is a critical mediator of the anti-inflammatory immune response. Adenosine is released in the infected tissue from astrocytes and invading immune cells. The binding of adenosine to adenosinergic receptors expressed on immune cells triggers a G-protein–mediated signaling cascade that alters the production of pro- and anti-inflammatory mediators (immune cytokines). Activation of ADORA2A, ADORA2B, or ADORA3 on macrophages, monocytes, and dendritic cells decreases pro-inflammatory mediators including IL-12, TNF-α, MIP-1α and increases the secretion of the anti-inflammatory IL-10 and VEGF (Haskó et al., 2005). *Abbreviations*: ADORA2A, adenosine A2A receptor; ADORA2B, adenosine A2B receptor; ADORA3, adenosine A3 receptor; IL-10, interleukin-10; IL-12, interleukin-12; MIP-1α, macrophage inflammatory protein-1α; TNF-α, tumor necrosis factor-α; VEGF, vascular endothelial growth factor.

A major environmental risk factor that has been extensively studied is the prenatal maternal exposure to viral or bacterial infections (Meyer, 2014). A viral infection can trigger the release of high amounts of ATP in cells of the innate immune system following identification of the virus (Burnstock and Boeynaems, 2014; Cekic and Linden, 2016). ATP then activates a cascade of pro-inflammatory events involving the activation of purinergic receptors located on the surface of immune cells (Abouelkhair, 2020). To limit the spread of the inflammation, adenosine counteracts the pro-inflammatory effect of ATP by acting on ADORA2A, ADORA2B, and ADORA3 expressed in the membrane of immune cells (Haskó et al., 2005; Abouelkhair, 2020; Franciosi et al., 2021; Figure 2). Degradation of ATP to adenosine by EctoNTs, such as CD39 and CD73, produces a surge in extracellular adenosine that stimulates nearby ADORA2A and ADORA2B to protect the infected host from excessive inflammatory tissue damage (Johnston-Cox et al., 2011; Di Virgilio and Vuerich, 2015; Franciosi et al., 2021). Adenosine acts as a brake that dampens the pro-inflammatory immune response *via* activation of ADORA2A and ADORA2B. Hence, drugs that increase extracellular adenosine concentration, such as dipyridamole, which inhibits adenosine reuptake, is a proposed therapeutic option to counter the hyperinflammation caused by COVID-19 infections (Kanthi et al., 2020). This could be achieved by enhancing the activity of EctoNTs, including CD39 and CD73, which is expected to shift the ATP/adenosine ratio to favor the anti-inflammatory action of adenosine, as suggested by Franciosi et al. (2021). Likewise, ADORA2A or ADORA2B agonists could be used to suppress the explosive inflammation caused by COVID-19 infections.

Adenosine augmentation during pregnancy may potentially protect the developing fetus from the increased risk for the resulting offspring to develop schizophrenia later in life. Adenosine augmentation may even be administered prophylactically when an imminent risk of an infection is expected such as during regional COVID-19 or flu outbreaks, especially amongst pregnant women with a family history of schizophrenia. However, excessive stimulation of ADORA2A and ADORA2B could weaken the maternal immune response to an extent that it fails to fight the infection. Any potential benefits against the detrimental effects of maternal immune activation must be balanced against the harms from excessive adenosine augmentation, resulting in an inverted U-shaped dependency on augmentation dosage that likely also varies from one pregnant woman to another.

On the other hand, if the activity of EctoNTs is downregulated in schizophrenia (Aliagas et al., 2013; O’Donovan et al., 2018), then the degradation of ATP to adenosine during an infection would be less efficient and the anti-inflammatory response to counter the pro-inflammatory action of ATP in early postnatal life would be weak. The immunosuppressant action of adenosine may be further weakened by the faster degradation of adenosine by ADA reported in schizophrenia (Dutra et al., 2010; Sasidharan et al., 2017; O’Donovan et al., 2018). Hence, lower adenosine production in schizophrenia may enhance the vulnerability to environmental risk factors in early post-natal life when the brain is undergoing rapid development.

Another environmental risk factor is perinatal hypoxia (Meyer and Feldon, 2010). Similar to a viral infection, hypoxia triggers an ATP release followed by a transient surge of extracellular adenosine as ATP is being broken down by EctoNTs. This may underlie the protection of ADORA1 knockout against brain developmental defect, i.e., ventriculomegaly, due to neonatal hypoxia (postnatal days 3–14) in comparison with wild type mice (Turner et al., 2003). Excessive ADORA1 activation therefore could be toxic for the immature brain. Likewise, activation of ADORA1 has also been linked to glycemia-induced neural injury in the developing brain (Kim et al., 2005).

Conversely, ADORA1^−/−^ knockout mice showed more severe brain abnormalities after neonatal hypoxic ischemic brain injury (Winerdal et al., 2016). In a rat model of neonatal hypoxic ischemia, ADORA1 antagonists administered during or after hypoxic brain injury did not alter the amount of brain damage but increased mortality when given before brain injury (Bona et al., 1997). Furthermore, in a mouse model of multiple sclerosis, ADORA1 deletion exacerbated axonal damage in the brain and spinal cord, and the exacerbated damage was associated with the up- and downregulation of pro- and anti-inflammatory gene expression, respectively (Tsutsui et al., 2004). The authors went on to suggest the potential use of ADORA1 agonists in neuroinflammatory diseases.

The paucity and conflicting data available do not permit one to easily conclude if ADORA1 stimulation is necessarily detrimental to the immature brain. Whether ADORA1 activation is protective or neurotoxic would likely be uncertain unless the immunological activation due to the early life insults can be well specified. This difficulty would argue against the use of adenosine augmentation in pregnant women during viral infection to minimize the risk of abnormal *in utero* and postpartum brain development in the offspring.

**Figure 3:**
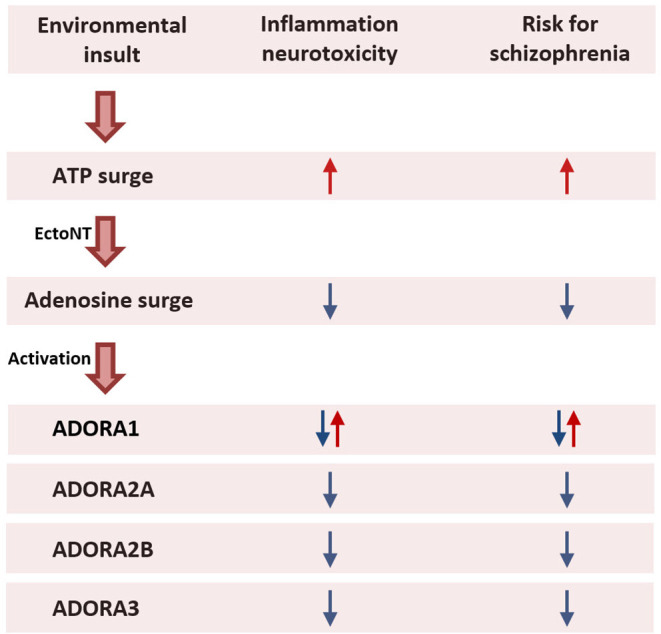
Prenatal maternal exposure to environmental insults. Exposure to an environmental insult during pregnancy such as a viral infection activates the maternal immune system that evokes a cascade of pro- and anti-inflammatory events. An initial surge in ATP activates pro-inflammatory agents to fight the infection. The increased production of ATP is followed by a secondary surge in adenosine production through enzymatic degradation of ATP by EctoNTs. Mediated *via* ADORA2A, ADORA2B, and ADORA3 located on the surface of immune cells, adenosine triggers an anti-inflammatory response to limit the spread of the inflammation and prevent excessive tissue damage (Haskó et al., 2005; Abouelkhair, 2020; Franciosi et al., 2021). The pro-inflammatory action of ATP disturbs fetal brain development and therefore increases the risk for the offspring to develop schizophrenia later in life. By contrast, the anti-inflammatory action of adenosine protects the fetal brain from the harmful effects of the inflammation. Consequently, augmenting adenosine in pregnant women during an infection may reduce the risk for schizophrenia in the offspring. While the anti-inflammatory action of adenosine is mediated *via* ADORA2A and ADORA2B and possibly ADORA3, it remains unclear whether activation of ADORA1 is protective or harmful to fetal brain development. *Abbreviations*: ADORA1, adenosine A1 receptor; ADORA2A, adenosine A2A receptor; ADORA2B, adenosine A2B receptor; ADORA3, adenosine A3 receptor; ATP, adenosine triphosphate; EctoNT, ectonucleotidase.

In this section, we characterize the final common pathway of infection, hypoxia, ischemia, and glycemia as early life environmental predisposing factors of schizophrenia as the initial pro-inflammatory ATP surge is followed by the anti-inflammatory counter surge in adenosine, which directly binds to adenosine receptors (ADORA2A, ADORA2B, and possibly ADORA3) on the surface of immune cells. In this scheme, the resulting pro-inflammatory ATP signal is considered detrimental to brain development, and against which adenosine would be viewed as protective (Figure 3).

### Adenosine deaminase acting on RNA as target against aberrant RNA editing in schizophrenia

In addition to ADA that is responsible for the deamination of free adenosine, a distinct family of deaminases has evolved whose substrate is double-stranded RNA (dsRNA), known as adenosine deaminase acting on RNA (Bass, 2002). In humans, there are two active variants: adenosine deaminase RNA specific (ADAR) and adenosine deaminase RNA specific B1 (ADARB1), which are implicated in diverse developmental processes including neurodevelopment (Shtrichman et al., 2012; Li and Church, 2013; Rajendren et al., 2021), and one inactive variant, adenosine deaminase RNA specific B2 (ADARB2) (Savva et al., 2012), which intriguingly shows a brain specific expression (Yang et al., 2021). While the *ADA* gene is located on the human chromosome 20 (Tischfield et al., 1974; Jhanwar et al., 1989), the *ADAR*, *ADARB1*, and *ADARB2* genes are located on chromosomes 1, 21, and 10, respectively (Samuel, 2011; Wang et al., 2017). ADAR, ADARB1, and ADARB2 can alter gene expression or amino acid sequence of the encoded protein by converting adenosine nucleosides to inosine in pre-mRNAs, mRNAs, tRNAs, and non-coding RNAs since the A-to-I conversation is read as guanosine by the splicing and translation machinery (Kleinman et al., 2012). Such post-transcriptional A-to-I editing events are crucial to development and function of the brain given that they are found in various genes expressed in the CNS (Yang et al., 2021). Alterations in coding regions of mRNA are rare while A-to-I conversions in noncoding regions can have epigenetic consequences.

Alterations in A-to-I RNA editing is known to be widespread in the brain of patients with schizophrenia (Breen et al., 2019). They are believed to be of pathophysiological significance and largely not attributable to exposure to antipsychotic medication. Although the causes for the altered RNA editing are not known, the possible involvement of ADAR enzymes has been highlighted by reports of dysregulated expression of *ADAR* genes in the schizophrenic brain. In post-mortem samples taken from schizophrenia patients, *ADAR* and *ADARB1* expression in the anterior cingulate cortex and DLPFC was upregulated (Silberberg et al., 2012; Breen et al., 2019), while *ADARB1* expression in the prefrontal regions was downregulated (Kubota-Sakashita et al., 2014) in comparison with healthy controls. Targeting the expression or enzymatic activity of ADAR and ADARB1 may therefore provide a novel strategy to control pathological RNA editing implicated in the etiology of schizophrenia. Admittedly speculative, it may be easily evaluated in etiological models of schizophrenia such as the maternal immune activation model (Meyer, 2014). Indeed, alterations in ADAR-mediated RNA-editing can be triggered by immune activation. Worth noting is that between the two isoforms of ADAR due to alternative splicing of the *ADAR* gene (George and Samuel, 1999), the 150-kDa isoform acts as an interferon-inducible antiviral effector that is expressed in the cytoplasm (Patterson and Samuel, 1995; George and Samuel, 1999; Poulsen et al., 2001).

In the established mouse model of prenatal exposure to maternal infection (PEtMI), pregnant dams are injected with the viral mimic, polyriboinosinic-polyribocytidylic acid, Poly(I:C), at a sensitive phase during gestation. When timed at around gestational day 9, the acute immune challenge reliably generates a wide spectrum of neuro-behavioral abnormalities in the offspring that closely resembles the brain and psycho-pathological disturbances reported in schizophrenia patients (Meyer, 2014). The same procedure also increased A-to-I RNA editing in fetuses harvested on gestational day 10, i.e., 24 h after the Poly(I:C) challenge to the pregnant dams (Tsivion-Visbord et al., 2020). Furthermore, the altered RNA editing was accompanied by an increased expression of the interferon-inducible 150-kDa isoform of ADAR in the fetal brains from the Poly(I:C)-treated dams. The selective upregulation of the 150-kDa isoform agrees with it being a downstream event to the interferon surge triggered by Poly(I:C). However, the increase in A-to-I RNA editing was no longer present in the offspring when they reached adulthood. It remains to be tested whether the transient increase in A-to-I RNA editing is causal for the subsequent emergence of schizophrenia-like brain and behavioral phenotypes (induced by the PEtMI model) at adolescent and adult age (Meyer, 2014). If so, it may lead to the possibility that a suitably timed intervention to suppress A-to-I RNA editing may nullify a significant early-life environmental risk of schizophrenia. The aberrant expression of neural editomes after the initial infection-triggered expression of interferon-inducible ADAR may be further associated with epigenetic events with long-lasting implications on the pathogenesis and progression of schizophrenia such as the intriguing observation of an association between high levels of interferon and treatment resistance, as well as exacerbation of negative symptoms in schizophrenia patients (Momtazmanesh et al., 2019).

### Adenosine as a regulator of DNA methylation—targeting epigenetic risks

DNA methylation is an epigenetic process that regulates gene expression. Alternations in the DNA methylation status have been observed in schizophrenia, and epigenetic modifications including DNA methylation are considered a risk factor for the disease (Magwai et al., 2021). DNA methylation is critically influenced by the ambient adenosine concentration (Williams-Karnesky et al., 2013). It has been verified in animals that higher ambient levels of adenosine are expected to inhibit DNA methylation while lower levels lead to DNA hypermethylation.

Williams-Karnesky et al. (2013) have demonstrated that elevation of ambient adenosine, either through local adenosine releasing polymer implants, downregulation of ADK expression, or systemic ADK inhibition, can all reverse DNA hypermethylation in the epileptic hippocampus. They went on to show that conversely, DNA methylation was upregulated by ADK over-expression that lowered ambient adenosine concentration. Notably, the suppression of DNA hypermethylation by adenosine augmentation treatment was long-lasting since the suppression persisted long after the augmentation therapy had ended (Williams-Karnesky et al., 2013). Although the causal mechanism underlying DNA methylation, gene expression, and development of schizophrenia is far from understood, it represents a potential strategy to modify the early progression of the disorder. This approach may be uniquely suitable for prodromal patients when mild symptoms may not justify the use of conventional antipsychotic drugs. The possibility that targeting DNA methylation may not require chronic treatment is another reason that this approach warrants further exploration. Here, we highlight the adenosine regulation as a potential mediator amenable for manipulation. Existing techniques include brain implants of adenosine releasing polymers and advanced gene therapies to achieve up- or down-regulation of the methylation of specific gene loci.

The ability to reliably achieve very precise epigenetic effects will be essential. As demonstrated in the PEtMI mouse model, genome wide changes in the DNA methylation status include hyper- as well as hypo-methylation depending on the genomic loci (Richetto et al., 2017). The pattern of the induced epigenetic changes further depends on the precise gestational day when Poly(I:C) is injected. As some of the methylation changes induced by Poly(I:C) treatment seem to be modifiable over time, it may be possible to prevent or even reverse methylation changes in patients or healthy subjects with histories of prenatal infections.

### Caffeine consumption during pregnancy—a significant risk of schizophrenia in the offspring?

Exposure to specific chemical risk factors may come in the form of food and drinks consumed by the mothers during pregnancy. Caffeine (1,3,7-trimethylxanthine) is a naturally occurring alkaloid that is regularly consumed through our diets (Heckman et al., 2010). It is habitually consumed as coffee, tea, and energy drinks worldwide including pregnant women (Onaolapo and Onaolapo, 2011; Chen L.-W. et al., 2014; Li et al., 2015). Despite the recommended safe level of daily consumption of about 200 mg, consumption during pregnancy at lower doses is associated with slower fetal growth (Gleason et al., 2021). Pregnant women may be more vulnerable to caffeine (Dlugosz and Bracken, 1992). The combination of an extended half-life of caffeine in pregnant females, poor fetal extraction rate, and rapid penetration of maternal caffeine into the placenta are all contributing factors. Exposure to caffeine in drinking water (75, 300, or 1,000 mg/L) in pregnant mice was sufficient to induce working memory deficits and potentiate locomotor response to acute cocaine in the resulting offspring upon reaching adulthood (Björklund et al., 2008; Soellner et al., 2009). Even perinatal exposure during lactation could modify depressive-like behavior of the offspring upon reaching adulthood, although female offspring appeared more vulnerable, and the effects were bi-directional depending on dosage (Laureano-Melo et al., 2016; Magenis et al., 2022). Nonetheless, DNA damage in the brain of adult offspring was observed regardless of sex, and thus strongly implicating that caffeine consumption during pregnancy could be detrimental to fetal brain development.

Björklund et al. (2008) suggested that an enhanced psychostimulant response induced by prenatal caffeine is largely mediated by ADORA1 blockade. They showed that heterozygous ADORA1^+/−^ knockout mice with partial loss of ADORA1 activity comparable to their prenatal caffeine model showed a similar psychotic-prone phenotype. Moreover, it appeared that the ADORA1 reduction in the maternal host, rather than that in the fetus, was responsible, because they observed that offspring with wild type genotype born to ADORA1^+/−^ dams also displayed the phenotype. Björklund et al. (2008) further identified epigenetic transmission of the psychotic-prone phenotype in wild type offspring whose mothers were also of wild type genotype, but the grandmothers were ADORA1^+/−^. Although the precise ADORA1-dependent epigenetic mechanism remains unclear, it illustrates that ADORA1 blockade during gestation could be a risk factor with transgenerational consequences. The adenosine hypothesis of schizophrenia may be extended to consider such etiological risk factors, which reinforces a multifaceted impact (neurodevelopment, immune imbalance, and epigenetics) of adenosine hypofunction from a life-long perspective. However, despite evidence from a large Danish national birth cohort study (*N* = 47,491) showing that maternal caffeine consumption during pregnancy is associated with an increased risk of developing psychiatric disorders in the offspring (Mikkelsen et al., 2017), more research is necessary to pinpoint the precise contribution of dietary caffeine intake during pregnancy as a risk of schizophrenia. Until such a link can be unequivocally demonstrated as causal, and the underlying mechanism sufficiently delineated to specify what may qualify as excessive caffeine consumption, it would be premature to attribute the maternal behavior in question as a risk of schizophrenia.

## Epilogue

Multiple contributing or modulating factors capable of reducing the ambient concentration of adenosine have been linked to schizophrenia in clinical as well as preclinical studies since the inception of the adenosine hypofunction hypothesis of schizophrenia two decades ago. The reviewed data suggest the need for further investigation and revision of the hypothesis, including its utilization in generating potentially novel antipsychotic drugs. We intend to illustrate in this review that the correspondence between adenosine efficiency and psychopathology is far from simple. This message should be clear even though we have not included any systemic evaluation of explorative transcriptomic and proteomic studies in schizophrenia. Alterations in the expressions of diverse biomolecules related to the adenosine system (broadly defined) would not be surprising (e.g., Kathuria et al., 2020), but we would anticipate that, while some findings may support the adenosine hypofunction hypothesis, there will likely be inconsistent findings. Our decision to omit reviewing such data should not distract from their importance (which certainly warrant a separate review), but only reflect our emphasis on investigations directly driven to test specific predictions of the adenosine hypothesis of schizophrenia.

It is therefore not too surprising, at least at this stage of development, that the hypothesized potential of adjunctive adenosine augmentation to improve antipsychotic efficacy has yet been successfully translated to the clinic. In this respect, it is particularly worth noting again that contrary to the central tenet of the adenosine hypothesis, animal models continue to suggest that inhibition of striatal ADORA2A activity may potentially alleviate not only negative symptoms, but also cognitive impairments, related to schizophrenia. It is consistent with the speculation that heavy caffeine consumption by schizophrenia patients may constitute a form of self-medication. The viability of this pharmacological approach, however, is likely hindered by the risk of exacerbating psychotic symptoms of the disease. It follows that the co-administration of ADORA2A antagonists and conventional antipsychotics with potent D_2_R antagonistic action may be a necessity in addition to the hypothesized extension of therapeutic scope and efficacy to be gained from this adjunctive treatment strategy. Whether such an approach could bring about realistic improvement in the functioning and quality of life to people living with schizophrenia can only be decided by further clinical trials.

While acknowledging that considerable efforts would be required to overcome the current impasse in achieving effective symptom-oriented treatment, we have also explored the critical role of adenosine as a modulator of early life environmental risk associated with maternal immune activation. Here, adenosine augmentation may be conceived as an innovative treatment concept targeting etiology rather than symptom suppression. Its potential use for pregnant women with severe acute infection to minimize long-term neurodevelopmental risk to the fetus may not be far-fetched given the recent concerns over the mental health of children born during the COVID-19 pandemic (Lins, 2021; Edlow et al., 2022). Adenosine and its regulatory enzymes could be in a unique position to confer protection against maternal inflammation, DNA hypermethylation, and aberrant RNA editing to ameliorate not only early environmental insults on brain development but also a range of other chronic deficiencies.

## Author contributions

The creation of this review was a joint effort of PS and BY who wrote the entire manuscript and created the figures included in this publication. Both authors approved the submitted version.
